# Health Decisions Under Uncertainty: The Roles of Conspiracy Beliefs and Institutional Trust

**DOI:** 10.3390/bs15040524

**Published:** 2025-04-14

**Authors:** Erga Atad

**Affiliations:** Lauder School of Government, Reichman University, Herzliya 4610101, Israel; erga.atad@runi.ac.il

**Keywords:** health behavior change, information processing, vaccination hesitancy, institutional trust, conspiracy theories

## Abstract

Research on vaccination hesitancy has been extensive, but the role of information processing in decision making still needs to be explored. The study examines the alignment between parents’ COVID-19 vaccination intentions and actual behavior, focusing on the impact of different kinds of information processing on the consistency or inconsistency of these behaviors. It analyzes parents’ reliance on health information sources, and education levels, with institutional trust, ability to critically evaluate conspiracy theories and scientific knowledge as moderators. A total of 1118 Israeli parents participated in digital surveys and were classified into the following 4 groups based on their initial vaccination intentions and actual behaviors: (1) consistent—pro-vaccine and vaccinated; (2) inconsistent—pro-vaccine but unvaccinated; (3) inconsistent—anti-vaccine but vaccinated; and (4) consistent—anti-vaccine and unvaccinated). The results show that consistent parents process information using system 1, i.e., heuristic information processing, reliance on health information sources, moderated by institutional trust and education. However, inconsistent parents used systems 1 and 2, namely heuristic–systematic information processing, influenced by knowledge of COVID-19 and the ability to assess conspiracy theories critically.

## 1. Introduction

Vaccination hesitancy has been a central issue in health psychology research, especially in health behavior (Callaghan et al., 2019; Damnjanović et al., 2018). The COVID-19 pandemic has led to a global increase in vaccination hesitancy, including in Israel. Initially, Israel’s vaccination campaign was remarkably successful, focusing on high-risk groups and expanding to the general population. However, hesitancy became more pronounced during efforts to vaccinate younger populations, including babies and young children (Gendler & Ofri, 2021; Rosen et al., 2021). Parents expressed concern about possible side effects, the lack of long-term data, and the necessity of vaccinating their children, whom they perceived as less vulnerable to severe COVID-19 outcomes (Seddig et al., 2022).

Numerous studies have focused on vaccination hesitancy. Most of these studies assess vaccination intentions or actual behaviors at a single time point, failing to capture how cognitive information processing affects decision making about vaccination intentions and behavior over time (Bruel et al., 2020; Kumar et al., 2016; Verelst et al., 2018). This study addresses this gap by examining Israeli parents’ initial intentions and eventual decisions to vaccinate their children against COVID-19 over time. It analyzes the entire process, from intention to action, providing a comprehensive understanding of how information processing influences health decisions and behaviors as they develop. Using the dual-system or two-minds hypothesis and the heuristic–systematic model (HSM) integrated into it, this paper explains how different types of information processing influence parents’ decisions to vaccinate their children (Kahneman, 2011; Tversky & Kahneman, 1973, 1974). The dual process and HSM frameworks include heuristic (system 1) processing that relies on cognitive shortcuts and peripheral cues, such as the credibility of health information sources, leading to quick and intuitive judgments. Parents who support or oppose vaccination (consistently pro-vaccine and vaccinated and anti-vaccine and unvaccinated) tend to rely more on this heuristic processing. This claim, supported by previous studies, indicates that exposure to contradictory health news, rather than one-sided information, lowers perceived credibility and reduces the likelihood of adopting recommended health behaviors. In such cases, confusion leads to motivated reasoning, resulting in defensive responses, causing individuals to ignore the information and resist behavioral changes (Chang, 2015; Nagler, 2014).

Systematic (system 2) processing involves a slower and more deliberate analysis of message content, including carefully evaluating health information and scientific knowledge. Systematic processing is more heavily used alongside heuristic processing by hesitant, inconsistent parents, pro-vaccine but unvaccinated parents, and anti-vaccine but vaccinated parents) (Botzen et al., 2022; Chaiken, 1980; Chaiken & Maheswaran, 1994; Kahneman, 2011; Liu et al., 2023; Tversky & Kahneman, 1973, 1974). As Funke (2010) argues, information processing is not a simple dichotomy or an isolated cognatic process, particularly in vaccine decision making. Instead, it is a continuum of complex cognitive processes that contains fast and slow thinking and integrates various elements, such as emotions, motivations, and environment.

To examine how parents process information and decide about COVID-19 vaccination, this paper explores their reliance on expert versus non-expert sources. This reliance is moderated by their education level and institutional trust, influencing their intention to vaccinate as well as their actual behavior. According to the authority heuristic, highly motivated individuals, such as parents who consistently support vaccines (consistently pro-vaccine and vaccinated), tend to rely on authoritative sources, like physicians and scientists, perceiving them as credible cues; therefore, they are likelier to use heuristic (system 1) processing (Koh & Sundar, 2010; Kumar et al., 2016; Maddux & Rogers, 1980; Sundar, 2008). Conversely, parents who consistently oppose vaccines (consistently anti-vaccine and unvaccinated) exhibit defense-motivated reasoning, selectively processing information using heuristic cues to defend pre-existing beliefs (Pierri et al., 2022) while ignoring contradictory information (Todorov et al., 2002). Gigerenzer and Gaissmaier (2011) and Gigerenzer et al. (2022) argue that heuristics are efficient cognitive strategies, allowing for quicker and more accurate decisions.

Institutional trust and education may moderate the relationship between information sources and vaccination by affecting whether the information will be evaluated based on peripheral causes, such as the source’s credibility, or whether individuals engage in a more systematic evaluation. Higher education helps to comprehend better health information, which increases the willingness to vaccinate (Šuriņa et al., 2022). Additionally, trust in government institutions reduces the impact of misinformation (Chen et al., 2022) and improves compliance with preventive behaviors (Antinyan et al., 2021).

According to the dual-processing model, individuals may not always need external validation, such as guidance from trusted sources, to believe in the safety and effectiveness of COVID-19-related behaviors when making decisions. However, people tend to rely more on trusted sources when they lack information or the ability to make informed judgments (Kikut-Stein, 2023). Consequently, having scientific knowledge plays a vital role in moderating health behaviors. While scientific knowledge alone may not lead to vaccination (Crowell & Schunn, 2015), higher literacy levels about COVID-19 and vaccines can increase the willingness to be vaccinated (Galanis et al., 2022).

Mistrust in institutions often contributes to the spread of conspiracy theories (Jovančević & Milićević, 2020). During the COVID-19 pandemic, misinformation and conspiracies proliferated and spread via unofficial information sources, such as social media or websites, reducing trust in the healthcare system and leading to vaccine hesitancy (Pierri et al., 2022). This reliance among individuals with low institutional trust led them towards confirmation bias, selectively interpreting scientific information that supported their existing beliefs and further strengthening their vaccine hesitancy (Béchard et al., 2024). However, hesitancy tends to be lower among more educated parents, who are better equipped to distinguish between credible sources and misinformation (Lee et al., 2022; Romer & Jamieson, 2023). This paper argues that education is crucial in moderating parents’ decision to vaccinate their children, as it enhances their ability to evaluate conspiracy theories critically (Allen et al., 2023; Iannello et al., 2022).

During the pandemic, parents were concerned about their children contracting COVID-19. Systematic processing (system 2) enables careful evaluation of the risks and benefits, often leading to greater vaccination acceptance. In contrast, heuristic processing (system 1) uses cognitive shortcuts, such as availability heuristics (Kahneman, 2011; Tversky & Kahneman, 1973, 1974), increasing perceived risk and reducing the intention to vaccinate (Liu et al., 2023). Trust in health institutions is essential for lowering concerns and strengthening vaccination intentions, thus can also impact the concern about various vaccination decisions.

This paper examined parents’ intentions and behavior to vaccinate their children using digital surveys of 1118 Israeli parents at 3 time points. This paper used a longitudinal study before, during, and after the primary vaccination campaign for 12-to-15-year-olds. The final sample of 456 parents was divided into the following 4 groups regarding their initial intention and actual behavior: (1) consistent—pro-vaccine and vaccinated (39.4%); (2) inconsistent—pro-vaccine but unvaccinated (17.6%); (3) inconsistent—anti-vaccine but vaccinated (27.9%); and (4) consistent—anti-vaccine and unvaccinated (15.2%).

The findings suggest that consistent parents primarily relied on system 1 heuristic cues, such as the authority heuristic with the use of information sources and the availability heuristic referring to concerns about their children contracting COVID-19. Education levels and institutional trust moderated these relationships. This contrasts with inconsistent parents, who, in addition to using system 1, i.e., heuristic processing, also engaged in system 2, i.e., systematic processing, drawing on their scientific knowledge and critical evaluations of conspiracy theories to change their initial vaccination decisions. The study provides valuable insights into strategies for addressing vaccine hesitancy in decision-making processes.

### 1.1. Dual Information Process Models and COVID-19 Health Behavior

Heuristic (system 1) and systematic (system 2) processes and HSM can be valuable perspectives to examine parental decision making regarding COVID-19 vaccination. System 1 can include heuristics-peripheral cues, such as the “availability heuristic”, where past experiences with COVID-19 influence judgment (Kahneman, 2011; Tversky & Kahneman, 1973; Chaiken, 1980). Accordingly, parents may evaluate the likelihood of being infected based on anecdotal knowledge and “rules of thumb” rather than statistical reasoning (Tversky & Kahneman, 1974). In contrast, system 2, as described by HSM, involves slower, more deliberate mental effort and attention when assessing message content (Kahneman, 2011; Chaiken, 1980). While these models offer insights into vaccine hesitancy, real-world decision making is rarely a dichotomy; it exemplifies complex cognition and includes motivational and emotional processes in information processing (Funke, 2010).

Gigerenzer and Gaissmaier (2011) and Gigerenzer et al. (2022) argue that heuristics are simple yet efficient cognitive strategies in decision making. They often simplify complex judgments by ignoring certain information, allowing for quicker and usually more accurate decisions than traditional “rational” models suggest. This viewpoint challenges the notion that heuristics are inherently insufficient and can cause errors (Gigerenzer & Gaissmaier, 2011; Gigerenzer et al., 2022).

### 1.2. Information Sources, Education, and Trust in Institutions

A growing body of research indicates that the sources from which individuals receive information can significantly shape their perceptions of vaccines. Relying on unofficial information sources, such as social media or websites spreading misinformation, can reduce trust in the healthcare system and lead to vaccine hesitancy (Pierri et al., 2022). This study examines how Israeli parents’ reliance on expert versus non-expert sources and their education level influence their COVID-19 vaccination intention and behavior.

According to the authority heuristic, messages from authoritative sources, such as physicians, scientists, and the Ministry of Health, are perceived as more credible due to heuristic cues, particularly among highly motivated individuals (Chaiken & Maheswaran, 1994; Koh & Sundar, 2010; Maddux & Rogers, 1980; Sundar, 2008). Parents relying on authoritative sources, which are defined as heuristic cues, are likelier to use heuristic (system 1) processing. However, they can also engage in systematic (system 2) processing moderated by institutional trust (Chaiken, 1980; Chaiken & Maheswaran, 1994; Koh & Sundar, 2010; Maddux & Rogers, 1980; Sundar, 2008). Reliance on authoritative sources and high institutional trust strengthen vaccine intention.

Furthermore, knowledge about COVID-19 and critical evaluations of conspiracy theories can increase vaccination intention, particularly among those with moderate reliance on authoritative sources (Sundar, 2008). That aligns with Funke’s view of cognitive complexity, which involves an interplay between automatic and controlled processes. However, reliance on non-expert sources, such as social media, blogs, and friends who lack scientific expertise, triggers system 1 heuristic processing, where messages are evaluated based on source reliability, particularly among individuals with low institutional trust and low motivation (Koh & Sundar, 2010; Todorov et al., 2002). This can maintain or even increase parents’ vaccine refusal.

In such cases, confirmation bias may drive individuals with low institutional trust to selectively interpret scientific information that reinforces their beliefs, further strengthening vaccine hesitancy (Béchard et al., 2024). Additionally, differences in motivation influence how consistently pro-vaccine and vaccinated and anti-vaccine and unvaccinated parents are engaged with information sources. Parents involved with the COVID-19 pandemic actively seek and process information to bridge the gap between their actual and desired confidence. This aligns with the “sufficiency principle”, which suggests that highly motivated individuals engage in systematic (system 2) processing when they perceive a lack of confidence to create balance (Chaiken, 1980; Chaiken et al., 1989; Todorov et al., 2002). Anti-vaccine parents may selectively use heuristic (system 1) processing or systematic (system 2) processing to confirm their prior positions while ignoring other contrary information (Todorov et al., 2002).

#### 1.2.1. Motivated Reasoning, Institutional Trust, and Vaccine Hesitancy

Consistently pro-vaccine and vaccinated parents engage in accuracy-motivated information processing, aligning their judgments with reality through systematic, heuristic, or dual processing. Conversely, consistently anti-vaccine and unvaccinated parents exhibit defense-motivated reasoning, selectively processing information to defend pre-existing beliefs (Chaiken et al., 1996). They can accomplish this by selectively using heuristic or systematic processing to verify their preferred prior positions while ignoring other contrary information (Todorov et al., 2002). This aligns with the motivated reasoning theory, where individuals employ heuristic (system 1) or systematic (system 2) processing to confirm their existing attitudes while dismissing contradictory evidence (Kunda, 1990).

Accuracy and defense-motivated reasoning are crucial in vaccine hesitancy. This mindset is often rooted in institutional trust in health and politics, which shaped public behavior during the COVID-19 pandemic. Individuals with high institutional trust tend to accept more vaccinations, as trust influences vaccine safety preparation and encourages positive attitudes (Paredes et al., 2021).

Research suggests institutional trust, especially in public health agencies, is a stronger predictor of vaccination compliance (Kar et al., 2023) than trust in political leaders (Badman et al., 2022). Institutional trust is also associated with lower COVID-19 mortality rates (Yuan et al., 2022), highlighting its critical role in shaping health behavior (Han et al., 2023).

This study examines how institutional trust moderates the relationship between reliance on COVID-19 information sources on vaccine decisions, with the hypothesis that parental motivation—whether accuracy-driven (high) or defense-driven (low)—shapes vaccine decisions through heuristic and systematic processing. When the motivation is accuracy, it can lead to both heuristic and systematic processing. In contrast, defense motivations typically favor heuristic processing, relying on peripheral cues—such as the message’s source. Institutional trust may moderate the relationship between vaccination and the use of information sources by affecting whether individuals evaluate information based on peripheral causes (like the source’s credibility) or engage in a more systematic evaluation.

#### 1.2.2. Education and Trust as Mediators in Vaccine Decision Making

Research has highlighted the complex relationship between institutional trust, education, and health behavior during the COVID-19 pandemic. Higher education enables individuals to better understand and follow health measures, leading to increased vaccine knowledge and a greater willingness to vaccinate (Šuriņa et al., 2022). Furthermore, education and trust in government and healthcare professionals are associated with more substantial vaccine acceptance. Trust in government institutions also reduces the effect of misinformation (Chen et al., 2022). Higher education fosters positive vaccine perceptions, including greater trust in the vaccine, regulatory bodies, and institutional information sources and increased willingness to vaccinate (Liu et al., 2023; Falcone et al., 2022). Institutional trust also improves compliance with preventive behaviors (Antinyan et al., 2021).

Thus, the first hypothesis hypothesizes that parental reliance on official versus unofficial information sources influences vaccine decision making on COVID-19 through the mediation of education and institutional trust.

**H1.**  *Reliance on official health information sources (compared to unofficial sources) will affect vaccination intention and behavior through the mediation of education and institutional trust*.

### 1.3. Information Sources and COVID-19 Scientific Knowledge

According to the dual-processing model, parents who strongly believe in the safety and effectiveness of COVID-19-related behaviors may not need external validation, such as the guidance of trusted sources, when making decisions. However, reliance on trusted sources increases when individuals lack information or the ability to make informed judgments (Kikut-Stein, 2023). Parents relying on unofficial sources, like social media, family, and friends, may have lower COVID-19 knowledge and cannot make informed decisions. Conversely, parents who rely on official sources, such as the government, doctors, and healthcare providers, may not need extensive COVID-19 knowledge to make informed decisions.

Studies indicate that parents who turn to healthcare professionals as their primary source of information are more likely to ensure their children are vaccinated than those who rely on the Internet or family members (Charron et al., 2020). For example, mothers who trust their pediatricians and consider them as their primary source of information are 2.47 times more likely to vaccinate their children than those who rely on informal sources (Ashfield & Donelle, 2020; Cohen et al., 2023; Eller et al., 2019). While many parents seek vaccine information online, the reliability of this content can vary significantly, with numerous websites promoting misinformation and controversial anti-vaccine viewpoints (Shoup et al., 2019).

The relationship between scientific knowledge and health behavior is complex. While having scientific knowledge alone does not always lead to health behavior (Crowell & Schunn, 2015), a meta-analysis suggests that higher levels of literacy about COVID-19 or vaccination correlate with a greater willingness to get vaccinated (Galanis et al., 2022).

Therefore, the second hypothesis hypothesizes that pre-existing beliefs about vaccine safety and reliance on official information sources may influence parental vaccination decisions. This influence could be moderated by scientific knowledge, which may be processed heuristically, systematically, or both.

**H2.**  *Reliance on official health information sources (compared to unofficial sources) will increase vaccination intention and behavior through the mediation of COVID-19 scientific knowledge*.

### 1.4. Conspiracy Beliefs and Critical Content Evaluations

Mistrust in institutions often contributes to the spread of conspiracy theories (Jovančević & Milićević, 2020), which, in turn, can reinforce extreme beliefs about COVID-19 conspiracies and further reduce trust in governmental responses (Georgiou et al., 2020). This lack of trust can result in vaccine refusal (Fieselmann et al., 2022), as conspiracy theories undermine confidence in government and public institutions (Uscinski et al., 2016). This paper asserts that education level is critical in parents’ decision to vaccinate their children, primarily because it enhances their ability to evaluate conspiracy theories critically. Studies have shown that parents with a higher level of education are better equipped to assess the credibility of information, including vaccine-related misinformation. This increased ability helps them reject conspiracy theories and fosters a greater willingness to vaccinate their children (Allen et al., 2023; Iannello et al., 2022).

Belief in vaccine-related conspiracy theories is strongly linked to vaccine hesitancy; however, hesitancy is reduced among more educated parents who can differentiate between credible sources and misinformation (Lee et al., 2022; Romer & Jamieson, 2023). Educating parents with critical thinking skills and scientific literacy enhances their ability to navigate complex information environments, fostering greater trust in medical experts and public health recommendations.

Thus, the third hypothesis hypothesizes that parents’ education level influences their decision to vaccinate their children against COVID-19 through the mediation of their ability to evaluate conspiracy theories critically.

**H3.**  *A higher level of education (compared to lower levels) will increase vaccination intention and behavior through the mediation of critically evaluating conspiracy theories*.

### 1.5. Parents’ Concerns About Their Children Being Infected by COVID-19

The dual-process framework, as outlined in systems 1 and 2 and the HSM, provides a valuable perspective for understanding how parents process risks and their concerns about their children contracting COVID-19. Systematic processing (system 2) involves careful and effortful evaluation of risks and benefits, often leading to increased acceptance of vaccination by emphasizing the advantages of immunization. In contrast, heuristic processing (system 1) relies on cognitive shortcuts, such as availability heuristics (Kahneman, 2011; Tversky & Kahneman, 1973, 1974), which can heighten perceived risk and reduce the intention to vaccinate (Liu et al., 2023).

Parents’ concerns about COVID-19 and uncertainties about vaccine safety often amplify these risk precautions, contributing to vaccine hesitancy (Chu & Liu, 2021; Cohen et al., 2024). Trust in health institutions and government agencies is essential for reducing concerns and strengthening vaccination intentions. This trust affects beliefs in the accuracy of vaccine information and reduces fear-based reactions (Seddig et al., 2022). Concern about COVID-19 and trust in healthcare institutions influence parents’ decisions to vaccinate their children (Humble et al., 2021). Parents with greater confidence in institutional responses and trustworthy vaccine recommendations are more likely to vaccinate themselves and their children (Brandstetter et al., 2021).

Furthermore, research suggests that vaccine-related decisions are mediated by anxiety and institutional trust, highlighting the need to address emotional and informational barriers to vaccination (Park & Avery, 2023). Conversely, distrust in institutions heightens fear and skepticism, frequently resulting in vaccine hesitancy (Humble et al., 2021).

Thus, the fourth hypothesis hypothesizes that systematic processing (system 2) improves perceived benefits and increases the likelihood of vaccination. In contrast, heuristic processing (system 1) raises concern about risks and decreases vaccination. Parents’ concerns about their children contracting COVID-19 will influence their vaccination decision, mediated by their level of institutional trust.

**H4.**  *A higher level of parents’ concerns about their children being infected with COVID-19 (compared to lower levels) will increase vaccination intention and behavior through the mediation of institutional trust*.

## 2. Methods

### 2.1. Design and Approach

The research was conducted in a three-stage longitudinal study to test the research questions. The initial survey (stage 1a) took place from 28 April to 6 May 2021, anticipating the FDA Emergency Use Authorization (EUA) vaccination approval for 12–15-year-olds on 10 May 2021. Stage 2 was administered on 13–21 July 2021, several weeks after the vaccine was approved in Israel (2 June 2021). Stage 3 was conducted in November 2021 to allow late adopters to decide whether to vaccinate their children. Participants were recruited using simple random sampling via the Qualtrics platform through a commercial panel provider (IPanel). The inclusion criterion was being a parent of a child between 12 and 15. Participants received compensation of up to NIS 20 (approximately USD 6) for completing each survey. The institutional ethics committee approved the study (2021-034, 2021-056, 2021-089).

### 2.2. Sample

In total, 1118 parents responded to the first survey (stage 1a) in which they were asked to rate their intention to vaccinate their teenage children on a 1–5 Likert scale. Over half of the parents, n = 644, indicated they would definitely vaccinate their children. Since the study was primarily focused on parents likely to change their minds, the “definitely will vaccinate” group in the sample (stage 1b) was limited to 50 randomly chosen respondents. There was no need to restrict the “definitely will not vaccinate” group, as it numbered fewer than 50 (n = 29, 6.3% in stage 1a). The original cohort was chosen to represent the demographics of Hebrew-speaking Israelis (one language was used for linguistic simplicity).

In stage 2, after the FDA emergency approval and during the vaccination campaign, the sample of 456 individuals from stage 1b was used to examine their actual vaccination behavior. Of these, 376 completed the questionnaire. The remaining 80 parents were excluded from the data, either because they had not completed the entire survey or because they did so in an unreasonable amount of time (less than 3 min). In contrast, the average time to complete the survey was 14.6 min. The final sample was relatively more secular and educated than the Hebrew-speaking population (Table 1).

Of these 376 parents, 196 had vaccinated their children and were not questioned further. Another 180 (39.5%) reported that they did not vaccinate and were contacted again after four months (stage 3). Of these 180, 131 parents responded to the final survey. No information was collected on why 49 parents declined to answer.

It is important to note that longitudinal studies can potentially include self-selection bias. The second and third study participants may have been included selectively, influencing the sample characteristics. The study incorporated a random sample selection to mitigate this tendency and initially compensated participants for each survey. To avoid affecting the results, the study’s true purpose was disclosed.

## 3. Research Tools and Measures

The digital questionnaires were developed based on the recommendations of the SAGE Working Group on Vaccine Hesitancy (SAGE, 2014) and additional studies listed under the measures section.

### 3.1. Measures

The study asked and analyzed the 456 respondents (from stage 2) considerations related to 7 different groups of variables in their intention to vaccinate their children. The variables were measured in one or more of the other surveys, but only surveys 1 and 2 were used, as survey 3 only included 131 parents (Table 2).^1^

(1) Demographic variables—gender, income, religiosity (secular, traditional, religious, orthodox), and education level (high school, undergraduate degree, graduate degree, etc.).

(2) COVID-19 scientific knowledge—COVID-19 scientific knowledge was measured based on the following three items: “What does the Pfizer COVID-19 vaccine contain?” (item appeared in all three surveys), “What can we find in serological test” (item appeared in the second survey), and “why do mutations influence more on the vaccine efficacy” (item appeared in both the second and third surveys) (α = 0.75, M = 3.78, SD = 2.19). One credit point was provided for a correct answer selected, zero credit points were provided for an incorrect answer, and a mean average score was calculated for each respondent. The questions were adopted from previous survey (Davidson Institute of Science Education, 2020).

(3) General scientific knowledge—general scientific knowledge was measured using the question, “Do antibiotics kill viruses and bacteria?” (yes/no). Correct answers received one point (M = 0.78, SD = 0.41). The question was adapted from the National Science Foundation Battery, which evaluates scientific understanding (National Science Board, 2020).

(4) Reliance on official versus unofficial health information sources—respondents were asked to select one or more information sources from a list of sixteen sources they could use while searching for health information. The list included official and relevant trained sources, such as physicians and scientists, and unofficial or untrained sources, such as friends and social media. One credit point was provided if an official/trained source was selected, while 0 points were provided for unofficial/untrained sources, and a mean average point was calculated for each respondent (M = 0.49, SD = 0.28). The question was adopted from a previous study (Crescitelli et al., 2020).

(5) Trust in institutions—measured based on a 7-point scale ranging from 1 (strongly disagree) to 5 (strongly agree) and modeled as the mean of the following five items: (1) Vaccine manufacturers take care of my health. (2) I have confidence in the medical system. (3) The government is affected by pressure from drug companies. (4) If scientists say a vaccine is safe to use, I believe them. (5) I trust the drug companies to make safe vaccines (α = 0.82, M = 2.29, SD = 0.97). The question was adopted from a previous study (Crescitelli et al., 2020).

(6) Parents concerned about their children being infected with COVID-19—the level of concern regarding the possibility of parents’ children contracting COVID-19 was evaluated using a scale from 1 (not concerned at all) to 5 (extremely concerned) and computed as an average for each child individually (M = 2.83, SD = 1.22). The question was adopted from a previous study (Crescitelli et al., 2020).

(7) Belief in conspiracy theories—respondents were asked to select one or more explanations from a list of 8 that they use when critically evaluating fake news about COVID-19. The list included several statements, such as “The information was published on a social network, and I do not trust social networks”; “I cross-referenced it with official sources like the Ministry of Health website”; “The content did not make sense”; “I consulted personally with a person with relevant training (for example, a doctor). One credit point was awarded for each statement selected, so the highest scores indicate a high level of critical content analysis. In contrast, lower scores indicate a higher tendency toward belief in conspiracy theories. (M = 0.80, SD = 0.81).

### 3.2. Statistical Analysis

To test the hypotheses, a series of chi-square tests and analyses of variance (ANOVAs) were conducted to examine group differences. In addition, one logistic regression analysis was performed separately for each parent group, including all study measures as predictors. Finally, mediation analyses were conducted to compare the different survey groups. All statistical analyses were performed using SPSS version 28, including the PROCESS macro (Hayes, 2022), which was used to estimate mediation models.

## 4. Findings

Some parents were consistent in their decision, but others were not. To differentiate between parents who changed their minds and those who were consistent, we split the sample into four parental groups according to the coherence between their initial intention in May and actual behavior later in 2021 (Table 3).

I. Consistently pro-vaccine and vaccinated—parents who reported their intention to vaccinate and did so in practice (39.4%).

II. Inconsistently pro-vaccine but unvaccinated—pro-vaccination parents who did not vaccinate. These parents reported they intended to vaccinate their children or were hesitant but did not do so in practice (17.6%).

III. Inconsistently anti-vaccine but vaccinated: anti-vaccination parents who did vaccinate. These parents reported they did not intend to vaccinate their children or were hesitant, but they did vaccinate in practice (27.9%).

IV. Consistently anti-vaccine and unvaccinated—parents who reported their intention not to vaccinate and did not vaccinate in practice (15.2%).

No significant demographic differences were found between the four parent groups regarding gender, religiosity, highest level of science education, and income, except for education when using a chi-square test. The education level was higher among parents who vaccinated their children (Table 4), the consistently pro-vaccination and vaccinated (I) (56.7%), and those who were anti-vaccine but vaccinated (III) (57.1%). Those groups were followed by the consistently anti-vaccination and unvaccinated parents (IV) (40.4%) and the inconsistently pro-vaccine but unvaccinated (II) (34.9%). A following one-way ANOVA Scheffe post hoc test found significant differences between groups I and II and between groups II and III (Table 4).

Much of the analysis was performed for each group to differentiate measures and variables affecting their intentions and behaviors. The four parent groups differed in their levels of (1) institutional trust, (2) reliance on official information sources, (3) knowledge about COVID-19, (4) general science knowledge, (5) concern about children being infected with COVID-19, and (6) beliefs in conspiracy theories (Table 2).

Institutional trust was significantly different (*p* < 0.001) between the four parent groups: it was the highest among the consistently pro-vaccine and vaccinated parents (I) (M = 3.36; SD = 0.77) and lowest among the consistently anti-vaccine and unvaccinated parents (IV) (M = 1.77; SD = 0.75), with the inconsistent groups demonstrating a moderate level of trust: inconsistently pro-vaccine but unvaccinated (II) (M = 2.87, SD = 0.86); inconsistently anti-vaccine but vaccinated (III) (M = 2.87; SD = 0.89) (Table 2). Post hoc tests found multiple differences between the groups.

The different groups’ reliance on official health information sources differed significantly (*p* < 0.05). Again, it was highest among the consistently pro-vaccination parents (I) (M = 0.52; SD = 0.29) and lowest among the consistently anti-vaccine parents (IV) (M = 0.39; SD = 0.29), with a post hoc test pointing to a significant difference. The inconsistent groups demonstrated a moderate tendency to use official sources: inconsistently pro-vaccine but unvaccinated (II) (M = 0.49, SD = 0.29); inconsistently anti-vaccine but vaccinated (III) (M = 0.48; SD = 0.26) (Table 2).

Knowledge about COVID-19 was similar between the four parent groups. Surprisingly, the measure of knowledge about COVID-19 demonstrated that among the two consistent groups, the anti-vaccination (IV) group had a slightly higher level of knowledge (M = 0.80, SD = 0.22) compared to the pro-vaccination group (I), which demonstrated a lower level of knowledge (M = 0.74, SD = 0.26). However, the inconsistent groups differed from each other: the parents who were anti-vaccine but ended up vaccinating (III) had higher levels of knowledge about COVID-19 (M = 0.80, SD = 0.21) than the group that was pro-vaccine but ended up not vaccinating (II) (M = 0.73, SD = 0.26). This cannot be explained by their level of general science knowledge, which did not differ significantly between the parents’ groups. Furthermore, group III had a lower level of general knowledge (M = 0.77, SD = 0.38) than group II (M = 0.75, SD = 0.41). However, among the two consistent groups, the pro-vaccine (I) group had the highest level of general knowledge (M = 0.80, SD = 0.38) compared to the anti-vaccine group (IV), which demonstrated the lowest level of general knowledge (M = 0.67, SD = 0.42).

Additionally, the parents’ concerns about their children being infected was significantly different (*p* < 0.001) between the parents’ groups: it was the highest among the consistently pro-vaccine parents (I) (M = 3.11, SD = 1.15) and lowest among the consistently anti-vaccine parents (IV) (M = 2.12, SD = 1.29), and these two groups were significantly different in the post hoc test. The inconsistent group that finally decided to vaccinate (III) had a high level of concern (M = 2.91, SD = 1.17), which was also higher than the inconsistent group that finally chose not to vaccinate (II) (M = 2.65, SD = 1.18) (Table 2).

Beliefs in conspiracy theories were significantly different (*p* < 0.05) between the parents’ groups: the of two groups that were vaccinated, the inconsistently anti-vaccination parents that vaccinated (III) had the highest level of critical evaluation of the information (M = 0.97, SD = 0.89), followed by the consistently pro-vaccine group (I) (M = 0.80, SD = 83). However, the two non-vaccinated groups demonstrated the lowest levels, i.e., the consistently anti-vaccine group (IV) (M = 0.70, SD = 65) and the inconsistently pro-vaccine but vaccinated group (II) (M = 0.63, SD = 0.71).

To test the research hypotheses, we first used four logistic regressions, one for each parent group, including all the measures detailed in Table 2. The variables that were included were (1) institutional trust, (2) reliance on official health information sources, (3) knowledge about COVID-19, (4) general science knowledge, (5) education level, (6) parents’ concerns about their children being infected with COVID-19, and (7) beliefs in conspiracy theories (see Table 5). Their significance will be described for each parent group separately:

I. Consistently pro-vaccine and vaccinated: The institutional trust was positively and statistically significant for this group of parents (b = 0.878 *p* < 0.001), and its odds ratios indicated a higher chance of 2.40 for parents to be consistent in their intention to vaccinate. In addition, the COVID-19 knowledge was negatively and statistically significant for this group of parents (b = −1.40 *p* < 0.005), and its odds ratios included a lower chance of 0.24 for parents to be consistent in their intention to vaccinate. However, parents’ general scientific knowledge, the use of information sources, education level, parents’ concerns about their children being infected by COVID-19, and beliefs in conspiracy theories and the regression model for all variables were not statically significant, R^2^ = 0.23 (Nagelkerke) = 0.17 (Cox & Snell), χ^2^(8) = 11.04, *p* > 0.05.

II. Inconsistently pro-vaccine but unvaccinated: Parents in this group were less affected by their level of education in their decision not to vaccinate their children, as it was negatively and statistically significant (b = −0.282, *p* < 0.05), with an odds ratio of 0.75. All other variables in the model and the regression model were not statically significant, R^2^ = 0.05 (Nagelkerke) = 0.03 (Cox & Snell), χ^2^(8) = 3.78, *p* > 0.05. Eventually, this group decided not to vaccinate. This contrasts with the second inconsistent parent group, which made the opposite decision.

III. Inconsistently anti-vaccine but vaccinated: The parents in this group were more likely to evaluate the information critically and, thus, less sensitive to believing in conspiracy theories (b = 0.313, *p* < 0.05), with an odds ratio of 1.3. This regression model was not statically significant, R^2^ = 0.05 (Nagelkerke) = 0.03 (Cox & Snell), χ^2^(8) = 2.60, *p* > 0.05.

IV. Consistently anti-vaccination and unvaccinated: Parents in this group were less affected by the institutional trust that was negatively statically significant (b = −1.70, *p* < 0.001), and its odds ratios indicated a lower chance of 0.181 for parents not to vaccinate. A similar negative statically significant trend was found for using official information sources (b = −1.52, *p* < 0.05), and its odds ratios indicated a lower chance of 0.219 for parents not to vaccinate. However, they were more affected by COVID-19 knowledge, as it was positively and statistically significant (b = 2.9, *p*< 0.05), and its odds ratios indicated a higher chance of 8.15 for parents to be consistent in their intention not to vaccinate. However, this model was not statistically significant at R^2^ = 0.44 (Nagelkerke) = 0.25 (Cox & Snell), χ^2^(8) = 10.7, *p* > 0.05.

The next step was to find mediated relationships between the variables in each group. Guided by our theoretical background, the study focused on the reliance on official health information sources (H1–H2), education (H3), and parents’ concerns about their children being infected with COVID-19 (H4) as independent variables. These independent variables were mediated by four prominent variables highlighted by the regression (Table 5), namely institutional trust and education (H1, H4), knowledge about COVID-19 (H2), and beliefs in conspiracy theories (H3). SPSS PROCESS macro (Hayes, 2022) was used to run Model 6 based on 5000 bootstrap samples for the first mediated research hypothesis (H1), Model 4 for the second and third (H2–H3) hypotheses, and Model 1 for moderating hypothesis 4 (H4), which was the case for each parent group.

I. Consistently Pro-Vaccine and Vaccinated: Vaccinated as Intended

**Figure 1:**
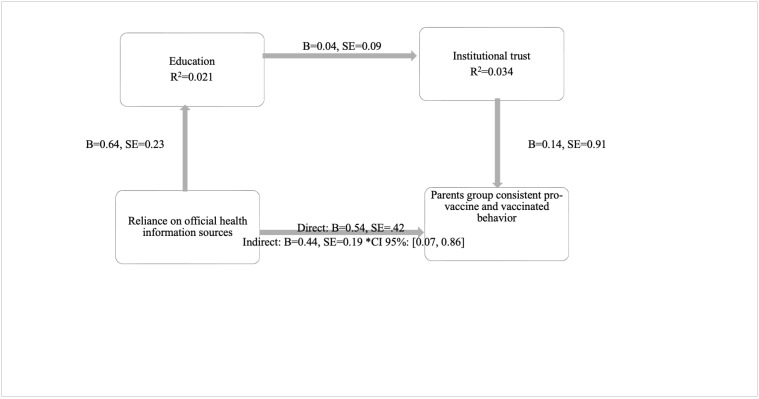
Parents’ consistent-pro-vaccine and vaccinated. Reliance on official health sources, directly and indirectly, affects vaccination behavior. Proposed Mediation Model (6) in SPSS Process Macro (Hayes, 2022) *.

H1 was tested to examine whether parents’ reliance on official health information sources would affect their pro-vaccination intention and behavior through the complete mediation of education and institutional trust. The total indirect effect of reliance on official health information sources on pro-vaccination behavior through education and institutional trust was significant (b = 0.44, SE = 0.20, CI 95%: [0.07, 0.86]). Parents who rely on official health information sources tend to have higher education levels, which, in turn, increases institutional trust. Higher institutional trust leads to increased pro-vaccination behavior. These findings highlight the critical role of institutional trust as a mediator and suggest that education strengthens trust in institutions, encouraging pro-consistent vaccination behavior (Figure 1).

**Figure 2:**
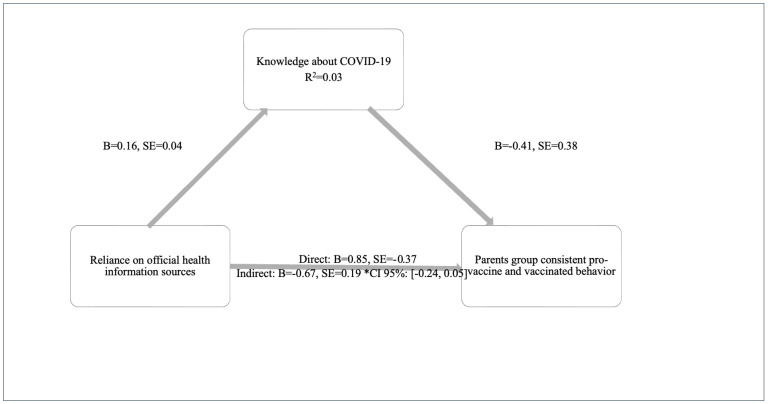
Parents’ consistent-pro-vaccine and vaccinated. Reliance on official health sources, directly and indirectly, affects vaccination behavior. Proposed Mediation Model (4) in SPSS Process Macro (Hayes, 2022) *.

H2 asked if parents’ reliance on official health information sources would affect their pro-vaccination intention and behavior through the complete mediation of knowledge about COVID-19. This knowledge did not mediate the parents’ reliance on official information sources and the consistently pro-vaccination relationship (b = −0.067, SE = 0.74, CI 95%: [−0.24 0.05]) (Figure 2).

**Figure 3:**
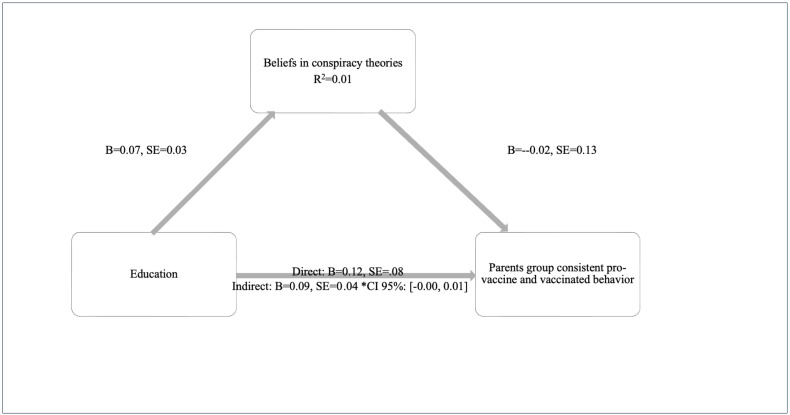
Parents’ consistent-pro-vaccine and vaccinated. Education, directly and indirectly, affects vaccination intention and behavior. Proposed Mediation Model (4) in SPSS Process Macro (Hayes, 2022) *.

H3 tested whether education would affect parents’ pro-vaccination intentions and behavior through the complete mediation of beliefs in conspiracy theories. Conspiracy beliefs did not mediate the parents’ education and the consistently pro-vaccination relationship (b = −0.00, SE = 0.01, CI 95%: [−0.02, 0.01]). Thus, conspiracy beliefs did not affect parents’ education in their consistently pro-vaccination behavior (Figure 3).

**Figure 4:**
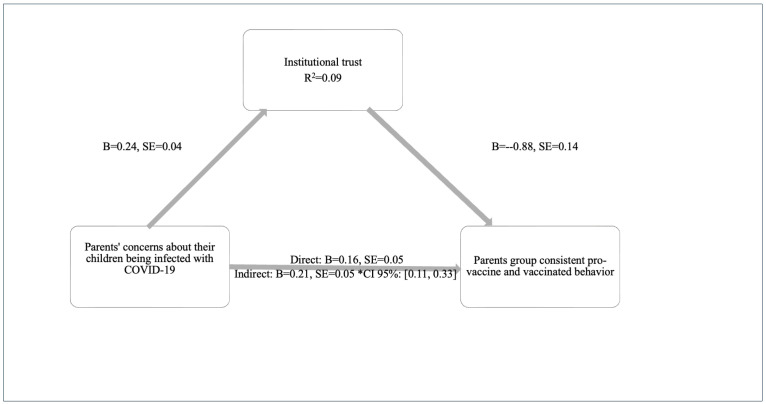
Parents’ consistent-pro-vaccine and vaccinated. Parents’ concerns about their children being infected with COVID-19 directly and indirectly affect vaccination behavior. Proposed Moderation Model (1) in SPSS Process Macro (Hayes, 2022) *.

H4 asked if parents’ concerns about their children being infected with COVID-19 affect pro-vaccination intention and behavior through the complete mediation of institutional trust. Parents’ institutional trust significantly mediated their concerns (b = 0.21, SE = 0.05, CI 95%: [0.11, 0.33]). This emphasizes the essential role that institutional trust plays in influencing parents’ vaccination decisions. When parents trust institutions more, their worries about their children contracting COVID-19 lead to intentions and behaviors supporting vaccination (Figure 4).

II. Inconsistently Pro-Vaccine but Unvaccinated: Intended to Vaccinate But Did Not

The mediation of reliance on official health information sources on pro-vaccine but unvaccinated behavior through education and institutional trust was insignificant (b = −0.20, SE = 0.13, CI 95%: [−0.49, 0.02]). However, education negatively significantly mediated the effect of reliance on official health information sources on vaccination behavior (b = −0.20, SE = 0.12, CI 95%: [−0.49, −0.02]) (H1). A mediation effect was also found with parents’ knowledge about COVID-19 did (b = −0.16, SE = 0.09, CI 95%: [−0.37, −0.00]) (H2). However, while they relied less on official information sources, their knowledge about COVID-19 was lower than the other groups, and they eventually decided not to vaccinate. These findings suggest that while reliance on official health information and institutional trust does not directly mediate, education and less knowledge about COVID-19 remain significant in negatively shaping vaccination behavior. This indicates that higher education reduced reliance on official health information sources and led to vaccination refusal. This is likely due to greater exposure to diverse, conflicting, and unofficial information sources, leading to skepticism about the vaccine, which results from lower COVID-19 knowledge. On the other hand, education did not mediate parents’ behavior through conspiracy beliefs (b = −0.02, SE = 0.01, CI 95%: [−0.07, 0.00]) (H3). In addition, the institutional trust did not significantly mediate parent’s concern about their children being infected with COVID-19 or the inconsistently pro-turned-anti relationship (b = 0.011, SE = 0.03, CI 95%: [−0.06, 0.08]) (H4). The absence of mediation through conspiracy beliefs and institutional trust highlights the complexity of factors influencing vaccine hesitancy behavior. (See the Supplementary Materials for Figures S5–S8: https://osf.io/pqx5a/?view_only=a4a7167533114ed79d1b27d62f4e0d41, accessed on 10 February 2025).

III. Inconsistently Anti-Vaccine but Vaccinated: Intended Not to Vaccinate But Did

The indirect effect of reliance on official health information sources on anti-vaccine but vaccinated behavior through education and institutional trust was insignificant (b = 0.08, SE = 0.09, CI 95%: [−0.09, 0.30]). However, education significantly mediated the relationship between reliance on official health information sources and vaccination behavior (b = 0.1204, SE = 0.0843, CI 95%: [0.0012, 0.3272]). Individuals who relied relatively on official health information sources had higher education levels, influencing vaccination behavior (H1). This may have also affected their knowledge about COVID-19 (b = 0.15, SE = 0.08, CI 95%: [0.01, 0.36]). As they relied on official health information sources, their knowledge about COVID-19 was relatively high, and they eventually decided to vaccinate (H2). Education also mediated their behavior through conspiracy beliefs (b = −0.02, SE = 0.01, CI 95%: [0.00, 0.06]) (H3). Higher education contributes to greater critical thinking skills, eliminates susceptibility to conspiracies, and contributes to the pro-vaccination decision. In addition, institutional trust did not significantly mediate these parents’ concerns about their children being infected with COVID-19 (b = −0.02, SE = 0.03, CI 95%: [−0.09, 0.03]) (H4). These results emphasize the pivotal role of education, knowledge about COVID-19, and critical thinking in shaping hesitant vaccination behavior and increasing vaccine uptake, especially for individuals who rely on official health information sources. (See the Supplementary Materials for Figures S9–S12: https://osf.io/pqx5a/?view_only=a4a7167533114ed79d1b27d62f4e0d41, accessed on 10 February 2025).

IV. Consistently anti-Vaccine and Unvaccinated: Did not vaccinate as intended

The indirect mediation effect of parents’ reliance on official health information sources influences vaccination behavior through the combined mediation of education and institutional trust was negatively significant (b = −0.80, SE = 0.38, CI 95%: [−1.64, −0.12]). The negative effects indicated that exposure to official sources mediated by the low institutional trust was associated with lower vaccination behavior, reflecting group-specific attitudes (H1). In other words, the official source could create the “boomerang effect”, negatively affecting the decision not to vaccinate. Also, knowledge about COVID-19 did not mediate the reliance on official information sources (b = 0.08, SE = 0.11, CI 95%: [−0.08, 0.35]) (H2). In addition, as these parents did not rely on official information sources and their education and institutional trust were relatively low, they were consistent with their decision not to vaccinate. That may also explain why education was not mediated through beliefs in conspiracy theories (b = −0.01, SE = 0.01, CI 95%: [−0.05, 0.01]) (H3). In addition, institutional trust, which was the lowest among the four parents’ groups, significantly negatively mediated these parents’ concerns about their children being infected with COVID-19 (b = −0.40, SE = 0.10, CI 95%: [−0.64, −0.22]) (H4). In other words, lacking trust emphasizes less concern about the virus. (See the Supplementary Materials for Figures S13–S16: https://osf.io/pqx5a/?view_only=a4a7167533114ed79d1b27d62f4e0d41, accessed on 10 February 2025).

## 5. Discussion

This study explores the impact of information processing on parental decision making regarding COVID-19 vaccination for their children. It examines the alignment between initial intentions and actual behaviors to identify factors driving consistent and inconsistent vaccination behaviors. It analyzed the following four parent groups: consistently pro-vaccine parents that vaccinated, anti-vaccine parents that did vaccinate, hesitant inconsistently pro-vaccine parents that did not vaccinate, and anti-vaccine parents that vaccinated. The first two groups initially intended to vaccinate their children or not and stuck to their decisions. In contrast, the other two groups had initially planned to vaccinate or not but later changed their decision to the opposite. Previous studies have regarded vaccination as a stable decision based on intention or behavior. This study examines vaccination as an evolution of cognitive information processing that includes intention and behavior over time.

The results indicate that consistently pro-vaccine parents that vaccinated intended to vaccinate and did so, relied on official sources, such as healthcare professionals and the Ministry of Health, and followed their initial intentions, moderated by their education and institutional trust. Conversely, consistently anti-vaccine parents who did not vaccinate originally intended not to and did not vaccinate relied on unofficial sources, such as social media and blogs, moderated by a lower level of education and lack of institutional trust. This supports the existing literature, which emphasizes the role of credible sources in shaping pro-health behaviors (Seddig et al., 2022; Liu et al., 2023) and the importance of institutional trust in complying with government guidelines (Kar et al., 2023; Badman et al., 2022; Yuan et al., 2022). However, misinformation from unofficial sources has been linked to increased vaccine hesitancy (Uscinski et al., 2016).

That aligns with the authority heuristic. Consistently pro-vaccine parents that vaccinated who are highly motivated perceive messages from health experts as more credible due to system 1 heuristic cues (Chaiken & Maheswaran, 1994; Koh & Sundar, 2010; Maddux & Rogers, 1980; Sundar, 2008; Tversky & Kahneman, 1973, 1974). According to Gigerenzer and Gaissmaier (2011) and Gigerenzer et al. (2022), such heuristics can simplify the decision process, allowing quicker, usually more accurate decisions than complex models. These heuristic cues can also influence System 2, systematic processing (Chaiken, 1980; Chaiken & Maheswaran, 1994; Kahneman, 2011; Koh & Sundar, 2010; Maddux & Rogers, 1980; Sundar, 2008). In contrast, consistently anti-vaccine parents that did not vaccinate and who have low motivation relied on non-expert health sources and triggered system 1 heuristic processing while primarily evaluating messages based on source reliability (Koh & Sundar, 2010; Todorov et al., 2002). Thia also sheds light on the influence of motivation types on information processing. Consistently pro-vaccine parents engage in accuracy-motivated information processing, aligning their judgments with reality. In contrast, consistently anti-vaccine parents adopt defense-motivated reasoning, selectively processing information to defend pre-existing beliefs.

Inconsistent parents were anti-vaccine parents who intended not to vaccinate but did, and pro-vaccine parents who intended to vaccinate but did not. They relied on moderate levels of official health sources. They were likelier to engage in system 2, i.e., systematic processing, using their COVID-19 knowledge to change their minds about vaccination. However, while the anti-vaccine vaccinated group had a high level of knowledge, the pro-vaccine unvaccinated group had a low level, which affected their final decision differently. The HSM explains this as a shift from heuristic to systematic processing to explain differences between beliefs and the available evidence (Chaiken et al., 1989). As they did not previously strongly believe in the safety and effectiveness of the vaccine, they required trusted sources when making decisions. The use of moderate official sources, however, required a higher level of COVID-19 knowledge to make effective decisions (Kikut-Stein, 2023).

This shift also helped inconsistently anti-vaccine parents who vaccinated overcome their initial hesitancy. Their high level of education, moderated by their skills in critically evaluating conspiracy theories and misinformation, helped them overcome their initial hesitancy. In contrast, the pro-vaccine parents who did not vaccinate had low levels of education and critical evaluation skills. They were more sensitive to misinformation and conspiracy theories, negatively affecting their vaccination intention. These findings align with previous studies, indicating that education improves critical thinking skills, essential for navigating complex information environments (Allen et al., 2023; Galanis et al., 2022; Iannello et al., 2022). During COVID-19, misinformation spread through unofficial information sources reduced trust in the healthcare system (Pierri et al., 2022), particularly among those with low institutional trust. That led to confirmation bias, i.e., selectively interpreting scientific information supporting existing beliefs, increasing vaccine hesitancy (Béchard et al., 2024; Atad & David, 2024).

Education also influences the decisions of both consistently pro- and anti-vaccination parents, moderated by their institutional trust. The pro-vaccination parents had high levels in both, while the anti-vaccination parents had lower levels in both. Higher education and trust in government and healthcare professionals are associated with a greater willingness to receive the COVID-19 vaccine. Trust also reduces the influence of misinformation (Antinyan et al., 2021; Chen et al., 2022; Liu et al., 2023; Falcone et al., 2022).

Additionally, consistently pro-vaccination parents were more concerned about their children becoming infected, also had a high level of institutional trust. This contrasts with consistently anti-vaccination parents, who had low levels in both. This aligns with system 1, the availability heuristic, which can assess the probability of being infected with COVID-19 and its implications (Kahneman, 2011; Tversky & Kahneman, 1973, 1974). Fear of COVID-19 and trust are critical predictors of vaccination behavior (Humble et al., 2021; Brandstetter et al., 2021). Credible information is crucial for consistently pro-vaccination parents with deep concern and trust to reduce fears, which can result in system 1 heuristics (Tversky & Kahneman, 1973, 1974; Lewis & Atad, 2023). Conversely, consistently anti-vaccination parents with low concern and trust may rely on heuristics to support their non-vaccination decision (Botzen et al., 2022; Hale et al., 1995; Averbeck et al., 2011).

Systems 1 and 2 and the HSM propose that inconsistently anti-vaccine parents that vaccinated use system 2 for systematic information processing, which evaluates risks and benefits and increases vaccine acceptance by enhancing perceived benefits. Conversely, inconsistently pro-vaccine parents who did not vaccinate use system 1 heuristic processing, which relies on cognitive shortcuts, to improve risk perception and reduce vaccination intentions (Kahneman, 2011; Liu et al., 2023; Tversky & Kahneman, 1973, 1974). Parents’ concerns about COVID-19 and uncertainty about vaccine safety amplify these concerns, contributing to vaccine hesitancy (Chu & Liu, 2021).

This study extends systems 1 and 2 and the heuristic–systematic model using COVID-19 vaccination decision making. Consistent parents use more system 1 heuristic processing, relying on information sources and concern, to match their intentions with their actual behaviors. Institutional trust and education modify these processes. However, hesitant–inconsistent parents use systems 1 and 2, i.e., heuristic–systematic processing, to change their initial intentions into final behaviors, critically evaluating COVID-19 knowledge and conspiracy theories, mediated education, and information sources. However, as Funke (2010) argues, information processing is not a dichotomy process, particularly in vaccine decision making. Instead, it is a continuum of complex cognitive processes that contains fast and slow thinking and integrates various elements, such as emotions, motivations, and environment (Funke, 2010).

The case of COVID-19 vaccination highlights the temporary nature of scientific knowledge and the importance of how individuals interpret scientific findings in shaping trust in health recommendations. When communicated as inconclusive or evolving, findings may trigger confusion, reinforce pre-existing beliefs, or increase susceptibility to misinformation. As Flemming et al. (2015) demonstrate, high self-efficacy does not guarantee critical engagement with scientific uncertainty; rather, it may foster overconfidence and reduce sensitivity to limitations in the evidence. Béchard et al. (2024) further show that even when scientific tentativeness is recognized—particularly by those who perceive themselves as experts—prior beliefs and personal experiences can still guide interpretation, likely through mechanisms of cognitive dissonance. These findings reinforce the need for science communication strategies that convey the existence of scientific consensus and balance transparency and confidence. Doing so may reduce perceived uncertainty and foster more informed, resilient decision making among diverse audiences.

This study emphasizes the importance of building and maintaining institutional trust and making accurate information easily accessible to the public through trusted sources by improving parents’ health literacy and critical thinking skills to evaluate misinformation. The study has several limitations. The sample consisted of Israeli parents, which limits its generalizability. It relied on self-reported data, which may be subject to social desirability bias. Other factors, such as political ideology and personal experiences with COVID-19, could influence vaccination behavior. Future studies could address these limitations. Moreover, they should examine how to design more effective interventions that target vaccine-hesitant populations, such as improving critical evaluation skills.

## Figures and Tables

**Table 1:** Demographic characteristics of the survey population.

Characteristic	Pre/Post-EUA Sample		Four Months into the Campaign, Sample		General Hebrew-Speaking Population in Israel *
	n	%	n	%	%
Gender					
Women	192	51	66	50.4	51
Men	184	49	65	49.6	49
Religiosity					
Secular	215	57.2	63	48.1	44.3
Traditional	77	20.5	28	21.4	33.6
Religious	60	16.2	28	21.4	11.5
Ultra-Orthodox	23	6.1	12	9.2	10.2
Highest level of education					
No high school matriculation certificate	46	12.2	22	16.8	28.4
High school matriculation certificate	75	20	27	20.6	21.3
Post-secondary program, without academic degree	65	17.3	22	16.8	15.6
Academic degree	190	50.5	60	45.8	34.6
Income **					
Below average	91	24.2	39	29.9	
Average	129	34.3	52	39.4	
Above average	147	39.1	40	30.7	
Total	376		131		

* Source—Israel, Central Bureau of Statistics population. ** Income by Israel central bureau of statistics population income report for 2020. https://fs.knesset.gov.il/globaldocs/MMM/e8316a79-c6ac-eb11-8124-00155d0af32a/2_e8316a79-c6ac-eb11-8124-00155d0af32a_11_18101.pdf, accessed on 11 November 2024).

**Table 2:** Explaining parents’ decisions to vaccinate their children based on their knowledge about COVID-19, general knowledge, reliance on health information sources, trust, concern, and beliefs.

	Parents Group		Parents Group		Parents Group		Parents Group		
	Consistently Pro		Pro But Unvaccinated		Anti But Vaccinated		Consistently Anti		
	M	SD	M	SD	M	SD	M	SD	F
Knowledge about COVID-19	0.74	0.26	0.73	0.26	0.8	0.21	0.8	0.22	F(3, 360) = 2.21
General science knowledge	0.80	0.38	0.77	0.38	0.75	0.41	0.67	0.42	F(3, 369) = 1.50
Reliance on official health information sources	0.52	0.29	0.49	0.29	0.48	0.26	0.39	0.29	F(3, 370) = 2.94 *
Institutional trust	3.36	0.77	2.87	0.86	2.87	0.89	1.77	0.75	F(3, 340) = 45.82 **
Parents’ concerns about their children being infected with COVID-19	3.11	1.15	2.65	1.18	2.91	1.17	2.12	1.29	F(3, 336) = 8.91 **
Beliefs in conspiracy theories	0.8	0.83	0.63	0.71	0.97	0.89	0.7	0.65	F(3, 372) = 2.74 *

* *p* < 0.05; ** *p* < 0.001. * Note that only 50 of the 644 parents responding “Definitely will vaccinate” pre-EUA were included in the sample (stage 1b).

**Table 3:** A comparison of the four parental groups regarding their initial intentions versus actual behavior.

Pre-EUA Intention	Post-EUA Behavior (Vaccinated: 253 (67.3%))	Post-EUA Behavior (Not Vaccinated: 123 (32.7%))
I definitely will vaccinate	I. Consistently pro-vaccine and vaccinated 39.4% (148)	II. Inconsistently pro-vaccine but unvaccinated 17.6% (66)
Probably will vaccinate		
I have not decided yet	III. Inconsistently anti-vaccine but vaccinated 27.9% (105)	
Probably will not vaccinate		IV. Consistently anti-vaccine and unvaccinated 15.2% (57)
I definitely will not vaccinate		

**Table 4:** Parents’ decisions to vaccinate their children by demographic characteristics.

	Parents Group Consistently Pro		Parents Group Pro-But Unvaccinated		Parents Group Anti-But Vaccinated		Parents Group Consistently Anti		Sig. Test (χ^2^)
	n	%	n	%	n	%	n	%	
Gender									
Women	74	50	31	47	53	50.5	34	59.6	n.s.
Men	74	50	35	53	52	49.5	23	40.4	
Religiosity									
Secular	91	61.5	36	54.5	59	56.7	29	50.9	n.s.
Traditional	30	20.3	36	16.7	59	25	29	17.5	
Religious	20	13.5	13	19.7	17	16.3	10	17.5	
Ultra-Orthodox	7	4.7	6	9.1	2	1.9	8	14.0	
Highest level of education									
No high school matriculation certificate	17	11.5	16	24.2	6	5.7	7	12.3	25.80 **
High school matriculation certificate	23	15.5	14	21.2	20	19	18	31.6	
Post-secondary program, without academic degree	24	16.2	13	19.7	19	18.1	9	15.8	
Academic degree	84	56.7	23	34.9	60	57.1	23	40.4	
Highest level of science education									
Junior high school	20	14	5	8.2	10	9.6	10	19.6	n.s.
High school: mandatory and elective science course	66	46.2	36	59	44	42.3	19	37.3	
Non-academic tertiary education or professional training	6	4.2	4	6.6	3	2.9	3	5.9	
Academic degree	51	35.7	16	26.2	47	45.2	19	37.3	
Income									
Below average	34	23.3	16	25.8	24	23.1	17	30.9	n.s.
Average	42	28.8	22	35.5	43	41.3	22	40.0	
Above average	70	47.9	24	38.7	37	35.6	16	29.1	

Significance marked: ** *p* < 0.01.

**Table 5:** Logistic regression of four groups of parents.

	Parents Group (Consistently Pro)		Parents Group (Pro But Unvaccinated)		Parents Group (Anti But Vaccinated)		Parents Group (Consistently Anti)	
	B	95% CI	B	95% CI	B	95% CI	B	95% CI
Knowledge about COVID-19 scale	−1.406 *	0.084–0.718					2.09 *	1.22–54.15
General scientific knowledge								
Reliance on official health Information sources							−1.52 *	0.056–0.85
Institutional trust	0.878 **	1.78–3.23					−1.70 **	0.10–0.30
Educational level			−0.282 *	0.58–0.96				
Parents’ concerns about their children being infected with COVID-19.								
Beliefs in conspiracy theories					0.313 *	1.02–1.83		

Parents Group (Consistently Pro)—R^2^ = 0.24 (Nagelkerke), 0.17 (Cox & Snell), χ^2^(8) = 11.04, *p* > 0.05. Parents Group (Pro But Unvaccinated)—R^2^ = 0.05 (Nagelkerke), 0.03 (Cox & Snell), χ^2^(8) = 3.78, *p* > 0.05. Parents Group (Anti But Vaccinated)—R^2^ = 0.05 (Nagelkerke), 0.03 (Cox & Snell), χ^2^(8) = 2.60, *p* > 0.05. Parents Group (Consistently Anti)—R^2^ = 0.44 (Nagelkerke), 0.25 (Cox & Snell), χ^2^(8) = 10.74, *p* > 0.05. * *p* < 0.05; ** *p* < 0.001.

## Data Availability

Data is contained within the article/Supplementary Material.

## Note

1 COVID-19 vaccines are based on mRNA technology. This relatively unknown platform may influence public trust, perceived risk, and interpretations of scientific uncertainty; this aspect was taken into account when designing measures across all three studies.

## Supplementary Materials

The following supporting information can be downloaded at: https://osf.io/pqx5a/?view_only=a4a7167533114ed79d1b27d62f4e0d41, accessed on 10 February 2025.
